# Serum Levels of N- and C-ERC/Mesothelin and Clinicopathological Factors in Mesothelioma Patients and Those without Mesothelioma

**DOI:** 10.14789/jmj.JMJ22-0042-OA

**Published:** 2023-04-14

**Authors:** AI KOYANAGI, KAZUNORI KAJINO, SHUKO NOJIRI, MASAAKI ABE, TOSHIYUKI KOBAYASHI, YOSHINOBU SUGITANI, LIANG YUE, NAOMI OHTSUJI, ATSUSHI ARAKAWA, TADASHI SATO, KAZUHISA TAKAHASHI, KENJI SUZUKI, AKIRA ORIMO, TAKASHI YAO, OKIO HINO

**Affiliations:** 1 Department of Pathology and Oncology, Juntendo University Graduate School of Medicine, Tokyo, Japan; 1 Department of Pathology and Oncology, Juntendo University Graduate School of Medicine, Tokyo, Japan; 2 Department of Human Pathology, Juntendo University Graduate School of Medicine, Tokyo, Japan; 2 Department of Human Pathology, Juntendo University Graduate School of Medicine, Tokyo, Japan; 3 Clinical Translational Science Center, Juntendo University Graduate School of Medicine, Tokyo, Japan; 3 Clinical Translational Science Center, Juntendo University Graduate School of Medicine, Tokyo, Japan; 4 Medical Technology Innovation Center, Juntendo University Graduate School of Medicine, Tokyo, Japan; 4 Medical Technology Innovation Center, Juntendo University Graduate School of Medicine, Tokyo, Japan; 5 Department of Respiratory Medicine, Juntendo University Graduate School of Medicine, Tokyo, Japan; 5 Department of Respiratory Medicine, Juntendo University Graduate School of Medicine, Tokyo, Japan; 6 Department of General Thoracic Surgery, Juntendo University Graduate School of Medicine, Tokyo, Japan; 6 Department of General Thoracic Surgery, Juntendo University Graduate School of Medicine, Tokyo, Japan

**Keywords:** mesothelioma, mesothelin, expressed in renal carcinoma (ERC), N-ERC, C-ERC

## Abstract

**Objectives:**

ERC/mesothelin is a glycosylphosphatidylinositol (GPI)-anchor protein expressed in mesothelioma. A precursor protein is cleaved by proteases and an N-terminal fragment (N-ERC) is extracellularly secreted. A remaining C-terminal fragment (C-ERC) is tethered on cellular membranes by the GPI-anchor, but C-ERC is also released after cleavage by proteases. We and other groups reported that serum N-/C-ERC levels are associated with stages of mesothelioma and suggested the possibility of their usefulness as diagnostic markers. However, the N-ERC level is also influenced by renal functions that are not directly associated with conditions of mesothelioma. It is not known whether other clinical factors influence serum N-/C-ERC values. Furthermore, their relationship to the amount of ERC/Mesothelin in mesothelioma is not yet validated. The objective of this study is to clarify the relationship of serum N-/C-ERC levels and the status of mesothelioma and several clinical factors.

**Materials and Methods:**

We analyzed relations of serum N-/C-ERC levels and ages, gender and other clinical factors in 522 patients without mesothelioma and examined their relation to the amount of ERC/Mesothelin in mesothelioma tissues in 13 mesothelioma cases.

**Results:**

Serum N-ERC levels were influenced by renal functions. On the contrary, those of C-ERC were not influenced by any clinical factors examined in this study and were significantly correlated with the amount of ERC/Mesothelin in mesothelioma.

**Conclusion:**

Although both markers are good indicators of treatment-responses in individual patients with mesothelioma, only C-ERC reflected the amount of ERC/Mesothelin in mesothelioma among multiple patients, possibly because N-ERC was influenced by renal functions.

## Introduction

Mesothelioma is an aggressive malignant disease arising from mesothelial cells that cover the surface of pleural, pericardial and peritoneal cavities, and is commonly associated with asbestos exposure^1)^. It is intractable to conventional therapies. Even in the latest clinical trials using immune checkpoint inhibitors such as of nivolumab or pembrolizumab, the progression free survival was approximately 6 months and the overall survival was approximately 18 months^2, 3)^. Unsatisfactory effects of these treatments are partly associated with the difficulty in early diagnosis of mesothelioma.

**Figure 1:**
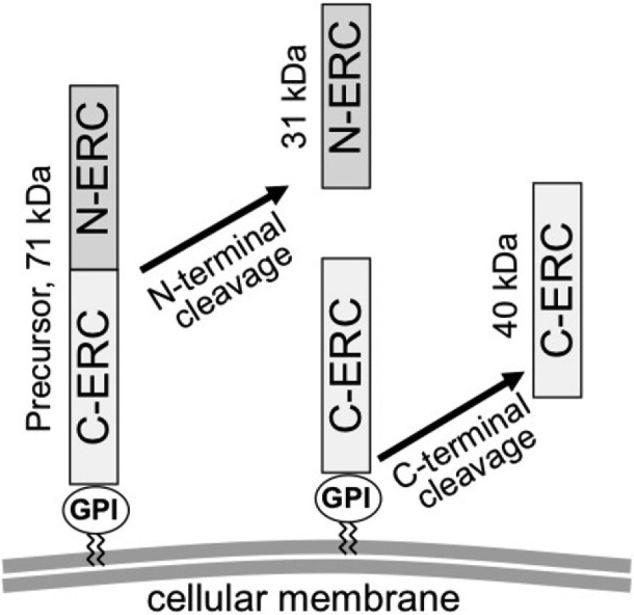
Structures of a 71-kD precursor ERC protein and two cleaved products, a 31-kDa N-ERC and a 40 kDa C-ERC. N-ERC and C-ERC are cleaved by proteases and released to the extracellular space. C-ERC, however, partially remains on the cellular membrane.

**Supplementary Figure 1:**
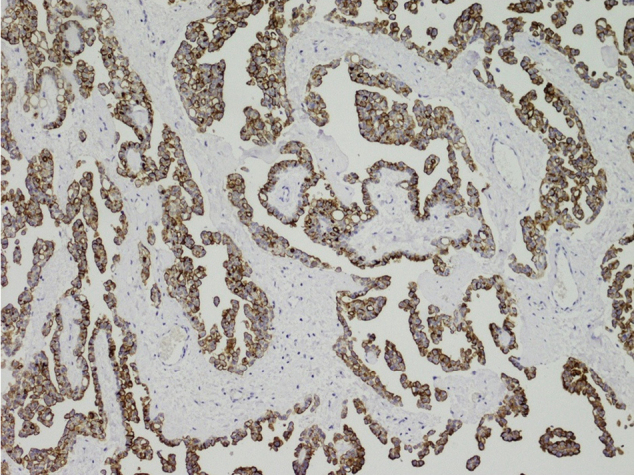
ERC expressed in mesothelioma tissue. ERC is detected by anti-C-ERC antibody on cellular membranes of epithelioid type mesothelioma.

Expressed in Renal Carcinoma (ERC) was originally isolated from renal carcinoma cells of Eker rat that hereditarily develops renal carcinoma^4)^, and ERC is a homologue of human Mesothelin (MSLN)^5)^. ERC/Mesothelin is a glycosylphosphatidylinositol (GPI)-anchor protein that is expressed on surface of normal mesothelium, epithelioid-type mesothelioma^6)^ or epithelioid components of biphasic mesothelioma. A 71-kDa ERC/Mesothelin-precursor protein is cleaved by proteases and a 31-kDa N-terminal fragment (N-ERC), that is identical to megakaryocyte potentiation factor (MPF)^7)^, is extracellularly secreted. A remaining 40-kDa C-terminal fragment (C-ERC) is tethered on cellular membranes by the GPI-anchor, but C-ERC is also released after incomplete cleavage by other proteases, and C-ERC partially remains on cellular membranes^8)^ (Figure 1, Supplementary Figure 1).

Asbestos/mesothelioma outpatient clinic was established in Juntendo University hospital in 2005, to screen the asbestos-exposed laborers and their family members for the early diagnosis of mesothelioma^9-10)^. The patients took the regular check of chest x-ray or blood test including N-ERC and C-ERC.

We and others previously reported that both N-ERC and C-ERC serum levels are increased in mesothelioma patients and suggested that they can be useful for the early diagnosis of mesothelioma, or indicators of the effectiveness of chemotherapy or surgical treatments^11-20)^. However, the relationship between their serum levels and the amount of ERC in mesothelioma tissue is not yet validated. Furthermore, Shiomi et al., reported that the serum N-ERC level is increased in patients with renal failure^21)^, and their findings suggested that it may be influenced by the clinical conditions that are not directly related to the status of mesothelioma. In this study, at first, we tried to clarify the relationship between the amount of ERC in mesothelioma tissue and the serum levels of N- or C-ERC. As a result, the serum levels of C-ERC were positively correlated to the amount of ERC in mesothelioma, but those of N-ERC were not. Secondly, because N-ERC is reported to be affected by the renal function, we examined whether the serum levels of N- or C-ERC are influenced by the clinical factors that are not directly associated with the mesothelioma. As a result, the serum levels of N-ERC were influenced by the renal function, as reported previously, but those of C-ERC was not. The C-ERC levels were not influenced by any of the clinical conditions examined in this study, other than those related to mesothelioma. Thirdly, we compared the clinical factors between the patients whose N-ERC was higher than C-ERC (N-higher group) and those whose C-ERC was higher than N-ERC (C-higher group). As a result, N-higher group included more women than men (p<0.05), although the reason for this phenomenon is to be clarified in the future.

Our study gave us a caution that we must be careful in the interpretation of serum C- and N- ERC values as the marker of mesothelioma among different patients. Both markers are, however, still valuable to monitor the status of mesothelioma in individual patients.

## Materials and Methods

### 1. Patients

Serum samples and biopsy or surgically resected specimens of mesothelioma were obtained from 42 mesothelioma patients and 522 outpatients who visited Asbestos/mesothelioma clinic in Juntendo University between 2005 and 2019. The patient characteristics in this study are shown in Table 1. None of 522 outpatients showed clinical evidence of mesothelioma. All procedures were performed in accordance with the Ethics Committee at Juntendo University School of Medicine (approval number: H05-0014) and with the Declaration of Helsinki. Written informed consent for participation in this study was obtained from all patients. As for the clinical course of mesothelioma patients during the chemotherapy, the clinical data was retrospectively obtained, and the effectiveness of treatment was evaluated by guidelines for response evaluation criteria in solid tumor (RECIST)^22)^.

**Table 1:**
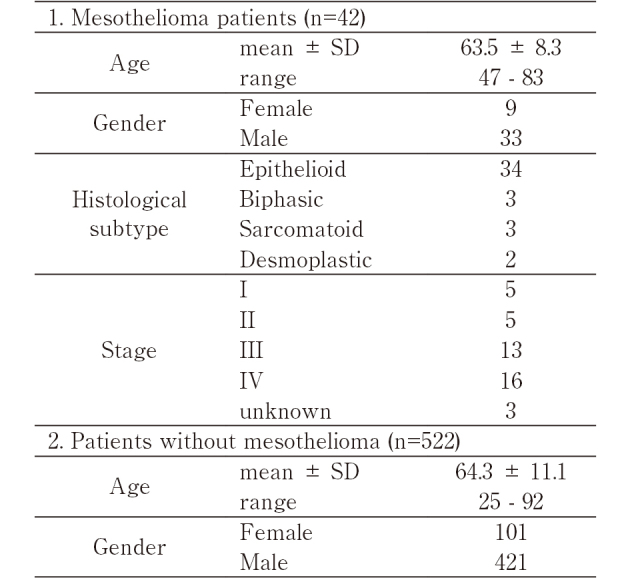
Clinical characteristics of patients

### 2. Immunohistochemistry of ERC in mesothelioma

To evaluate the expression status of ERC in mesothelioma tissue, we performed immunohistochemistry of mesothelioma tissue by using mouse monoclonal anti-C-ERC antibody 22A31 (#10357, Immuno-Biological Laboratories (IBL), Fujioka, Gunma, Japan) as a primary antibody. Tissue sections with 4-μm thickness were prepared from formalin fixed, paraffin embedded specimens of mesothelioma obtained by surgical resection or biopsy. After deparaffinization, the tissues sections were heated in 10mM citrate buffer (pH 6.0) for antigen retrieval and treated with 3% hydrogen peroxide. They were blocked with 5% normal goat serum and incubated with 2μg/mL 22A31 in Tris-buffered saline with 0.1% Tween 20 (TBS-T) at room temperature for 180 minutes. After washing with PBS-T, the specimens were incubated with EnVision+ System-HRP labeled polymer conjugated to goat anti-mouse immunoglobulins (DAKO K4001, Agilent Pathology Solutions, Santa Clara, CA, USA), at room temperature for 60 minutes. Finally, the slides were incubated with 3,3-diaminobenzidine (DAB) at room temperature for 3 min. Two pathologists observed findings and evaluated the percentage of ERC-positive area that was expressed as ERC-positive rate (%).

### 3. Enzyme-linked immunosorbent assay (ELISA) of serum N-ERC or C-ERC

Serum levels of N-ERC and C-ERC were determined by sandwich ELISA systems described previously^12, 23)^ with some modifications. Briefly, as for detection of N-ERC, two anti-N-ERC antibodies, monoclonal antibody (MoAb) 7E7^11)^ and horseradish peroxidase (HRP)-conjugated MoAb 16K16^12)^ were used as capture- and detection-antibodies respectively. As for detection of C-ERC, two anti-C-ERC antibodies, polyclonal antibody (PoAb) anti-C- ERC6^23)^ and HRP-conjugated PoAb anti-C-ERC^23)^ were used as capture- and detection-antibodies respectively. Dilution buffer for serum and detection antibody was 1% bovine serum albumin (BSA) in phosphate-buffered saline with 0.05% Tween 20 (PBS-T). Washing buffer was PBS-T. Diluted serum 100μL was loaded on each well coated with a capture-antibody and incubated at 37°C for 1 hour. After washing, 100μL of detection-antibody solution was added and incubated at 4°C for 30 minutes. After washing, for colorization, 100μL of tetramethylbenzidine solution (#19903, Immuno- Biological Laboratory, Fujioka, Gunma, Japan) was added and incubated at room temperature for 30 min in the dark place. Color development was stopped by 100μL of 1 N H_2_SO_4_. Absorbance of the solution at 450nm was measured in an ELISA reader (E-Max, Molecular Devices, Sunnyvale, CA, USA). The concentration of N- or C-ERC was determined by the standard curve derived from purified N- or C-ERC proteins^12, 23)^. Because of the outlying values of C-ERC in cases 28 and 294, and of N-ERC in a case 220 shown in Table 2, these three cases are removed from the further analyses.

**Table 2:**
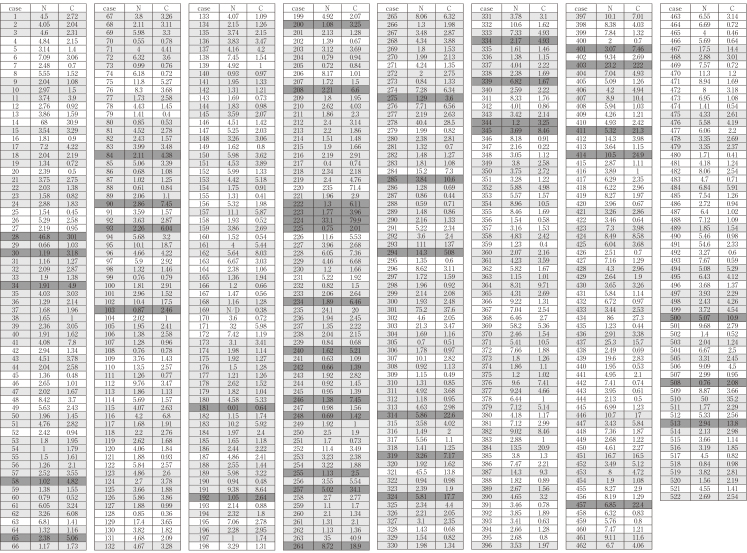
Serum levels [ng/mL] of N-ERC (N) and C-ERC (C) in 522 patients without mesothelioma C-higher patients (C > N x 2) are shown in dark gray, N-higher ones (N > C x 2) are shown in white, and the others are shown in light gray background. N/D, not determined (case 169). Note that Cases 28, 220 and 294 are removed from the further analysis because of outlying values.

### 4. Definition of N-higher and C-higher patients

As shown in Tables 2 and 3, some patients showed N-ERC higher than C-ERC, and the others showed the opposite tendency. We defined N-higher patients whose N-ERC values were more than twice higher than C-ERC, and similarly we defined C-higher patients showing C-ERC more than twice higher than N-ERC. In Tables 2 and 3, C-higher patients are shown in dark gray, N-higher ones are shown in white, and the others are shown in light gray backgrounds. Then we examined the relationship of N- or C-higher status and the clinical parameters.

**Table 3:**
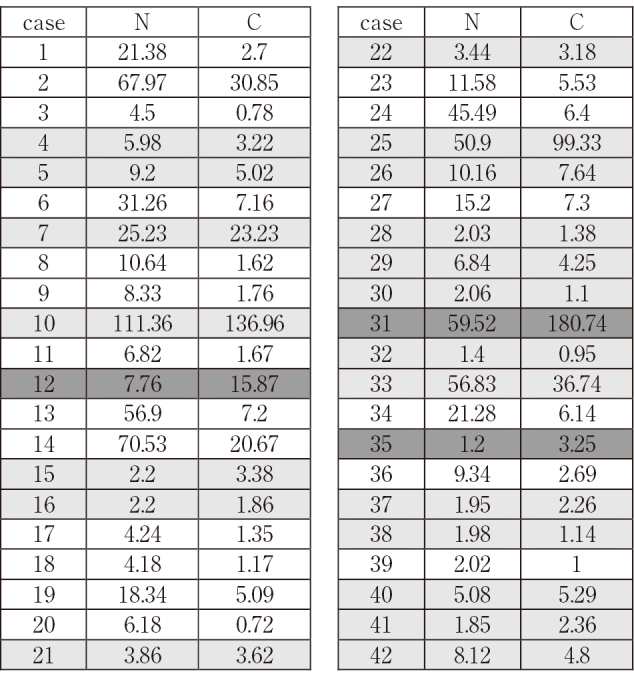
Serum levels [ng/mL] of N-ERC (N) and C-ERC (C) in 42 mesothelioma patients C-higher patients (C > N x 2) are shown in dark gray, N-higher ones (N > C x 2) are shown in white, and the others are shown in light gray.

### 5. Quantification of the ERC-positive volume of mesothelioma

We tried to clarify the relationships between the amount of ERC expressed in mesothelioma and the serum levels of N- or C-ERC. As an indicator of the amount of ERC protein in mesothelioma, we defined the ERC-positive volume (mL), as shown in the following formula.

ERC-positive volume (mL) = mesothelioma volume (mL) x ERC-positive rate (%)

Mesothelioma volume was calculated in 13 patients whose image data of mesothelioma were available by CT or macroscopic pictures of surgical specimens. These 13 cases included 11 epithelioid- and 2 biphasic-types. By using ImageJ software^24 25)^, we set region of interest (ROI) by drawing the circumference of mesothelioma on CT axial image of a 5-mm thick slice and calculated the area of ROI. The area (mm^2^) was multiplied by thickness (5 mm) to induce the volume (mL) of mesothelioma in each slice, and they were summed up to create tumor volume (mL) of whole mesothelioma. In cases in which the macroscopic pictures of surgical specimens were available, ROI was drawn around mesothelioma in each slice. Volume of whole mesothelioma was calculated by a same way as in the cases with CT images. Then we calculated ERC- positive volume (mL) by multiplying mesothelioma volume and ERC-positive rate (%) based on the results of immunohistochemistry in mesothelioma.

### 6. Clinical data

We evaluated the relationships between the serum levels of N- or C-ERC and clinical factors, such as age, gender and status relating to anemia (Hemoglobin [g/dL], Hb), inflammation (C-reactive protein [mg/dL], CRP), liver damages (alanine aminotransferase [IU/L], ALT), kidney function (Creatinine [mg/dL], Cre), nutrition (Albumin [g/dL], Alb), platelets count ([x104/µL], Plt), and diabetes (Hemoglobin A1c [%], HbA1c).

### 7. Statistical analysis

All data were analyzed with SAS version 9.4 (SAS Institute, Cary, NC, USA). To compare serum N-/C-ERC levels between mesothelioma patients and those without mesothelioma, t-test was used. Pearson’s correlation coefficient test was used to examine the correlation of serum N-ERC levels and C-ERC levels, serum N-/C-ERC levels and the ERC-positive volume in mesothelioma, and serum N-/C-ERC and clinical parameters. We considered that two factors are positively correlated when r > 0.40 and p < 0.05. Further, serum levels of N-/C- ERC between male and female, and clinical parameters between N-higher and C-higher patients were analyzed by t-test. The relationship between gender and N-/C-higher status was analyzed by chi-square test. A value of p < 0.05 was considered statistically significant.

## Results

**Figure 2:**
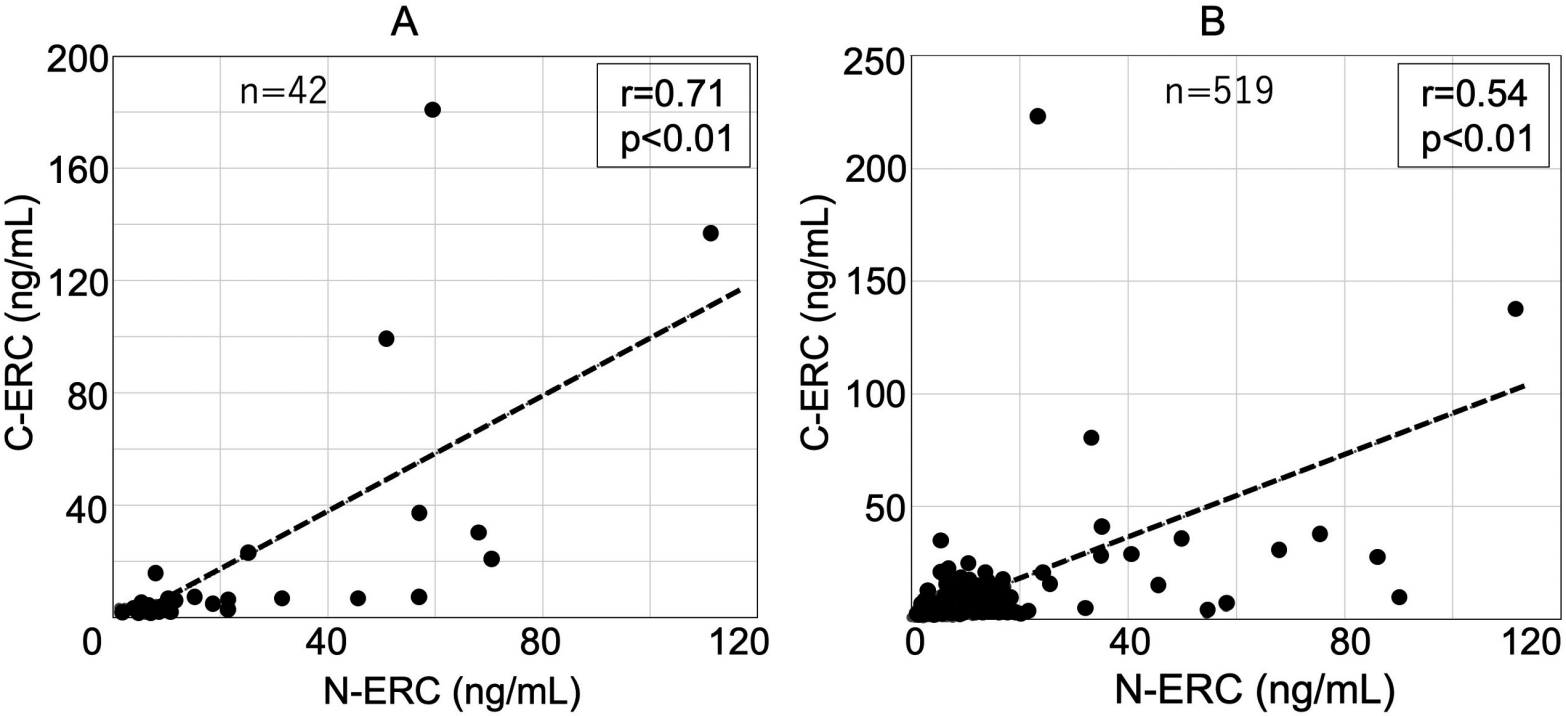
Relationship between serum N- and C-ERC in 42 mesothelioma patients (A) and 519 outpatients without mesothelioma in asbestos/mesothelioma clinic (B). Serum levels of N-ERC were positively correlated to those of C-ERC both in mesothelioma patients (A) and those without mesothelioma (B).

As expected, serum levels of N- and C-ERC were higher in mesothelioma patients than in those without mesothelioma (N-ERC; 19.0 ± 24.7 [n = 42] vs 5.4 ± 9.5 [n = 518], p < 0.05, C-ERC; 15.7 ± 36.2 [n = 42] vs 3.8 ± 8.4 [n = 518], p < 0.05). N- and C-ERC levels were positively correlated both in mesothelioma patients (Figure 2A) and those without mesothelioma (Figure 2B), with correlation coefficients 0.71 and 0.54 in mesothelioma patients and those without mesothelioma, respectively.

**Figure 3:**
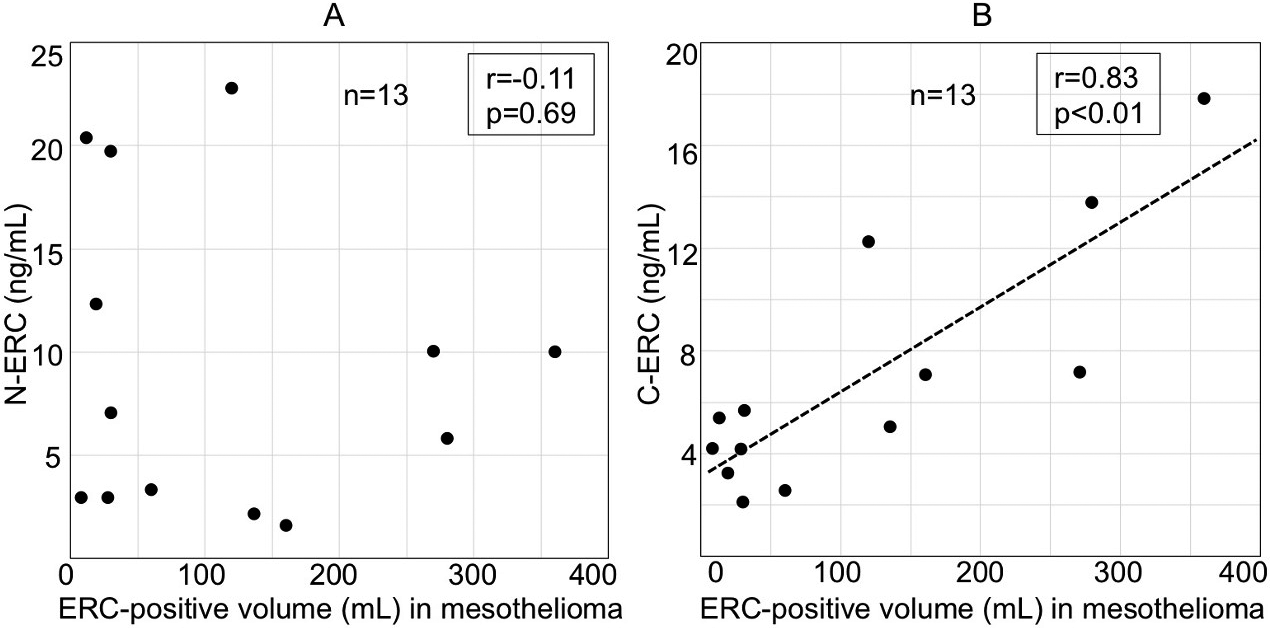
Relationship between ERC-positive volume (mL) in mesothelioma and serum levels of N-ERC (A) and C-ERC (B). Serum levels of C-ERC correlated to ERC-positive volume (mL) in mesothelioma (B), but that of N-ERC did not (A). ERC-positive volume (ml) was calculated by multiplying tumor volumes (mL) and ERC-positive area (%) in mesothelioma. n=13.

In 13 mesothelioma patients whose image data of the lesion was available, we checked the correlation between the serum levels of N- or C-ERC and the amount of ERC in mesothelioma that is expressed as the ERC-positive volume (mL). Serum levels of C-ERC correlated positively to the ERC-positive volume in mesothelioma (Figure 3B), but those of N-ERC did not (Figure 3A). Their raw data is shown in Supplementary Table 1.

**Figure 4:**
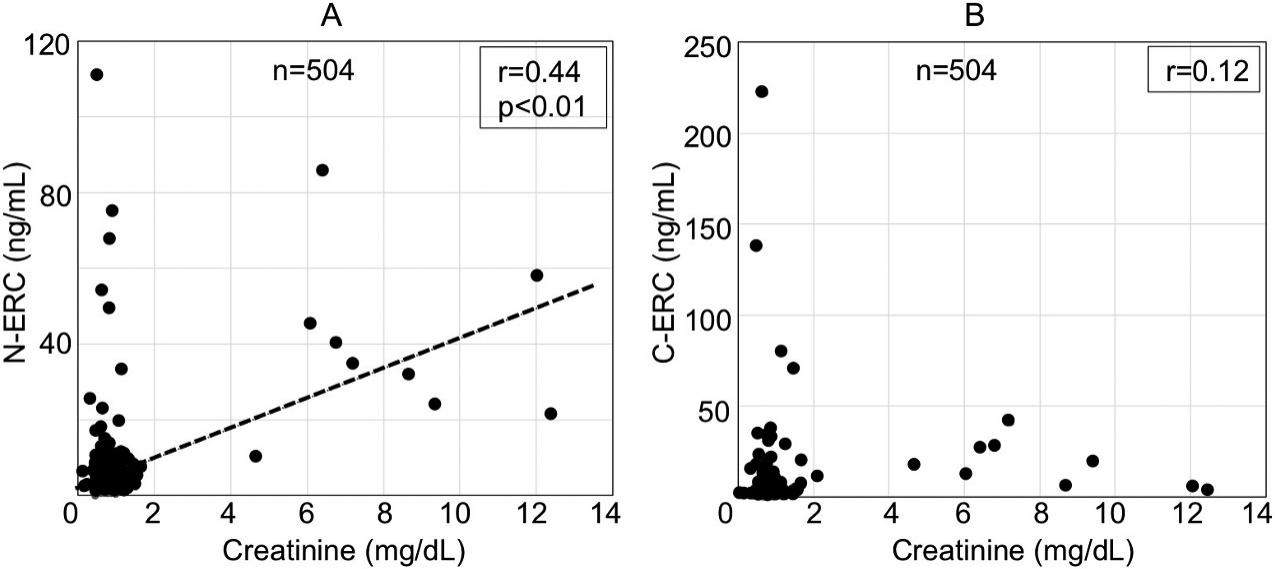
Relationship between serum levels of creatinine and N-ERC (A) or C-ERC (B) in 504 patients without mesothelioma. Serum levels of creatinine was positively correlated to N-ERC.

**Supplementary Figure 2:**
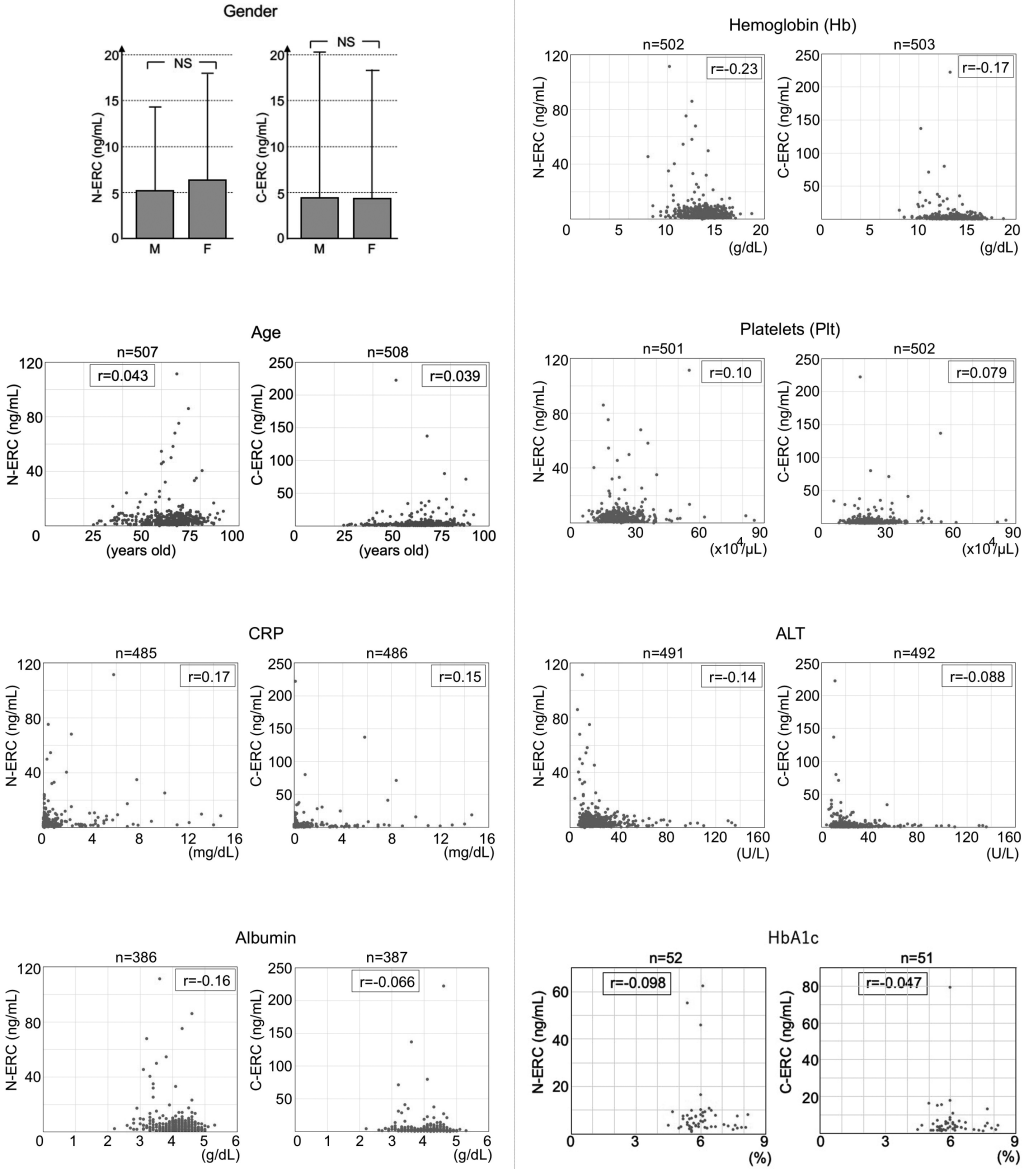
Relationship between serum levels of N- or C-ERC and clinical factors in patients without mesothelioma. Gender ; To compare N-/C-ERC levels between male and females, t-test was used. There was no significant difference of N-/C-ERC between male and female. NS, not significant. Age, CRP, Albumin, Hemoglobin, Platelets, ALT, HbA1c; Pearson's correlation coefficient test was used to examine the correlation of these parameters and serum N-/C-ERC levels. No correlation was observed between N-/C-ERC and these parameters. We considered that two factors are positively correlated when r > 0.40 and p < 0.05.

In patients without mesothelioma, we examined the relationship between the serum levels of N- or C-ERC and the clinical parameters such as age, gender and status relating to anemia, inflammation, liver damages, kidney function, nutrition, platelets count, and diabetes, as described in Materials and Methods. As a result, serum creatinine levels, as the marker of renal function, correlated positively to serum levels of N-ERC (Figure 4A), but not to those of C-ERC (Figure 4B). All the other clinical parameters examined in this study did not have any correlation to either of C- or N-ERC (Supplementary Figure 2).

Serum levels of N-/C-ERC in 522 patients without mesothelioma and 42 mesothelioma patients are listed in Tables 2 and 3, respectively. In some patients C-ERC was higher than N-ERC, and in the others N-ERC was higher than C-ERC. As described in Materials and Methods, we defined C-higher (C > N x 2), N-higher (N > C x 2) and the other patients, and they are shown in dark-gray, white, and light-gray backgrounds respectively in Tables 2 and 3. In 522 patients without mesothelioma, the numbers of C-higher and N-higher patients were 43 and 184, respectively. Between these two groups, we compared age, gender and the other clinical parameters. Results showed that no relationship was detected between N- or C-higher states and all clinical factors (Table 4) except for gender. More females than males were included in N-higher group with statistical significance (Table 5).

**Table 4:**
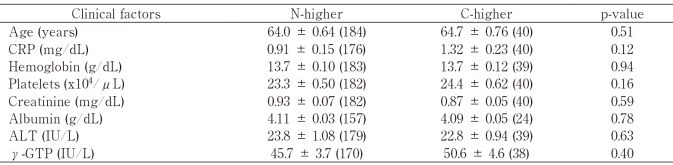
Clinical factors and N- or C-ERC higher status in patients without mesothelioma N-higher; N > C x 2. C-higher; C > N x 2. Number of cases are shown in parenthesis.

**Table 5:**
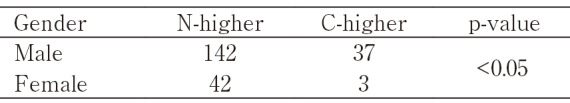
Gender and N- or C-ERC higher status in patients without mesothelioma N-higher; N > C x 2. C-higher; C > N x 2.

**Figure 5:**
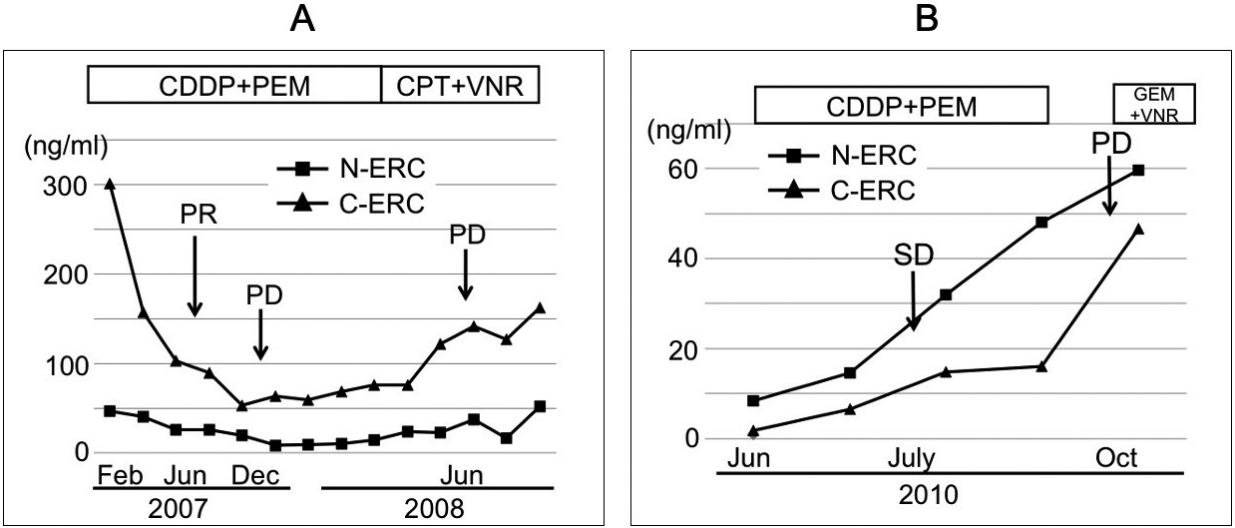
Changes of the serum levels of N- and C-ERC in two mesothelioma patients. Both of N- and C-ERC reflected the tumor burden of mesothelioma in individual patients. (A) The case showing C-ERC consistently higher than N-ERC, (B) The case showing N-ERC consistently higher than C-ERC. PR; partial response, PD; progressive disease, SD; stable disease, CDDP; Cisplatin, PEM; Pemetrexed sodium hydrate, CPT; Camptothecin (Irinotecan hydrochloride hydrate), VNR; Vinorelbine detartrate, GEM; Gemcitabine hydrochloride.

Figure 5 shows the changes of serum N- or C- ERC in 2 mesothelioma patients who received chemotherapies. Response rates to therapies as indicated by RECIST^16)^ system are also indicated. Both N- and C-ERC were shown to be reliable markers to reflect the tumor burdens in these two cases, as reported previously by other groups^14 16)^.

## Discussion

In mesothelioma tissues, ERC is localized on cellular membranes (Supplementary Figure 1), and it is detected by anti-C-ERC antibodies. On the contrary, anti-N-ERC antibodies^11, 12, 26)^, that recognize the internal amino acid sequences of N-ERC and detect N-ERC in the extracellular fluid, cannot make specific signals in any cellular components of mesothelioma (data not shown). These findings suggest that almost all N-ERC molecules are released into the extracellular spaces, and that C-ERC are partially released but some of them are remaining on the cellular membrane. Therefore, the amount of ERC in mesothelioma is evaluated by IHC using anti-C-ERC antibodies.

Creaney et al.^16)^ previously reported that serum levels of C-ERC correlated with volume of mesothelioma, and their work supports our result shown in Figure 3B. They, however, just measured volume of mesothelioma and they did not count the expressional state of ERC in mesothelioma. We compared the serum levels of N- or C-ERC and three parameters related to ERC in mesothelioma tissue: (A) tumor volume (ml), or (B) ERC-positive rate (%), or (C) ERC-positive volume [(C) = (A) x (B)]. All three parameters are defined in Materials and Methods. As shown in Supplementary Table 2, serum C-ERC level more significantly associated with ERC-positive volume (ml), than ERC-positive area (%). In our study, the significant relationship was not observed between serum C-ERC and tumor volume, as reported by Creaney et al.^14)^ possibly because of small number of cases (n=13) in our study. Serum level of N-ERC did not have any significant relationships with these three parameters.

Shiomi et al.^21)^ previously reported that serum levels of N-ERC are increased in the patients with renal failure. Therefore, in this study, we examined whether N- or C-ERC is influenced by renal functions and by the other clinical factors such as age, gender, inflammatory status, nutritional condition, anemia, liver function, platelets numbers, or diabetes. As shown in Figure 4, serum creatinine level positively correlated with N-ERC, but not with C-ERC, and this result is compatible with the report by Shiomi et al^21)^. Both of N- and C-ERC did not have any correlation to the other clinical factors examined in this study (Supplementary Figure 2). N-ERC is identical to megakaryocyte potentiation factor (MPF)^7)^ that has the activity to simulate megakaryocyte development in murine system^27)^, although the similar activity has not been reported in human. We studied the relationship between N-ERC and platelets numbers, and we did not find the correlation between them. Shiomi et al.^12)^ also reported that serum levels of N-ERC is influenced by age. In our data, the elder patients tended to have higher levels of N-ERC, but the correlation was not significant (Supplementary Figure 2).

Multiple studies reported that serum levels of N-ERC^11-14, 17-18, 20)^ and C-ERC^15-16, 19)^ reflect the tumor burden of mesothelioma. After the effective chemotherapy or surgical treatments, the serum levels of N-ERC^14)^ and C-ERC^16)^ decrease, and similar results are shown in Figure 5A. These findings indicated that both N- and C-ERC are good markers to monitor the status of mesothelioma in the clinical course of the same patients. Our present data (Figure 3) showed that, among different patients, C-ERC reflected the amount of ERC in mesothelioma more accurately than N-ERC, partly because N-ERC was influenced by renal function that is not directly associated with condition of mesothelioma.

It was puzzling for us why some patients showed that N-ERC was higher than C-ERC, and the others showed the opposite tendency. We tried to identify the causes of these phenomenon, but we were not able to find them, except for that N-higher patients included more female than male with statistical significances (Table 5). Renal clearance rate is generally higher in men than in women^28)^. If N-ERC is excreted through renal routes, the higher clearance activity of men can explain our finding that N-higher group included more female. This explanation is compatible with the data that serum levels of N-ERC showed a tendency to be higher in female than in male, although there was no statistical significance (Supplementary Figure 2). In a patient shown in Figure 5A, C-ERC was always higher than N-ERC, and the relationship was opposite in a patient in Figure 5B, and these data suggested the possibility that N-higher or C-higher states were determined by some factors intrinsic to each patient. Recently, sex differences in carcinogenesis are being discussed^29)^, and some hormonal condition may influence N-higher or C-higher states, although they are to be elucidated in future.

In conclusion, our study gave us a caution that we must be careful in the interpretation of serum N- and C-ERC values as the marker of mesothelioma among different patients, because N-ERC was influenced by renal functions that are not directly associated with conditions of mesothelioma. These markers are, however, still very valuable to monitor the status of mesothelioma in the same patients.

## Funding

AK was supported by Tsunagu-project in Advanced Educational Support Network for Young Pathology Doctors by the Ministry of Education, Culture, Sports, Science and Technology of Japan (MEXT). This study was supported in part by grants from Shizuoka Medical Research Center for Disaster of Juntendo University Shizuoka Hospital, and from the Institute for Environmental and Gender-Specific Medicine of Juntendo University Urayasu Hospital. This work was also supported by a Grant-in-Aid for Special Research in Subsidies for ordinary expenses of private schools from The Promotion and Mutual Aid Corporation for Private Schools of Japan.

## Author contributions

AK collected the data, undertook immunohistological studies, and drafted the manuscript. KK, TK, and YS designed this study. SN, AK, and KK performed statistical analysis. LY assisted the analysis of images to calculate the ERC-positive volume of mesothelioma. AA supervised the histological studies. MA performed ELISA to measure serum N- and C-ERC. NO assisted the immunohistochemical study. TS, KT, and KS supported the planning of this study, and AO, TY, and OH supervised this project. KK revised the manuscript. All the authors have read and approved the final version of this manuscript.

## Conflicts of interest statement

The Authors declare that there are no conflicts of interest.

**Supplementary Table 1:**
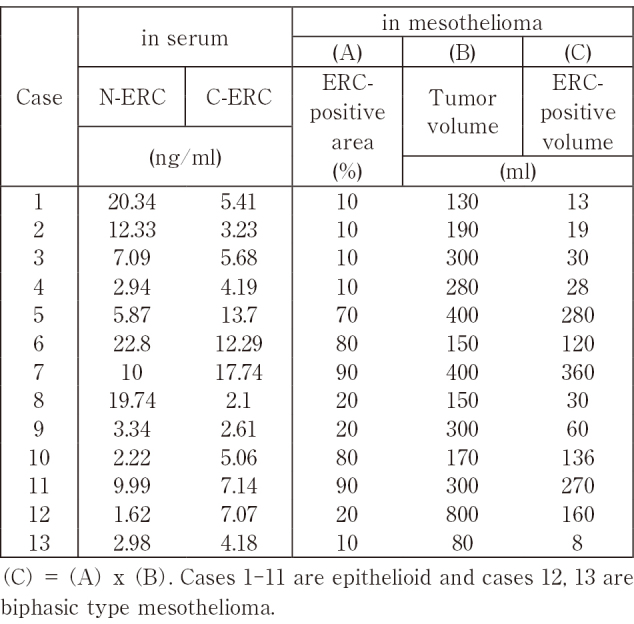
Relationship between N- or C-ERC levels in serum and the expression of ERC in mesothelioma

**Supplementary Table 2:**
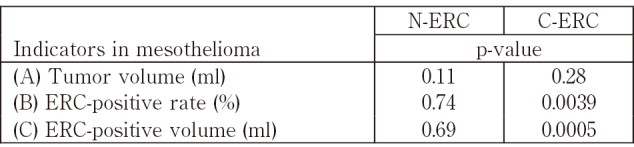
Relationships between N-ERC or C-ERC levels in serum and three indicators (A), (B), (C) in mesothelioma (C) = (A) x (B). Serum C-ERC is more significantly associated with ERC-positive volume (C) than ERC-positive rate (B) or Tumor volume (A). Serum N-ERC is not significantly associated with any of three indicators.

## References

[1] Robinson BWS, Lake RA: Advances in malignant mesothelioma. N Engl J Med, 2005; 353: 1591-1603. 16221782 10.1056/NEJMra050152

[2] Alley EW, Lopez J, Santoro A, et al: Clinical safety and activity of pembrolizumab in patients with malignant pleural mesothelioma (KEYNOTE-028): preliminary results from a non-randomised, open-label, phase 1b trial. Lancet Oncol, 2017; 18: 623-630. 28291584 10.1016/S1470-2045(17)30169-9

[3] Okada M, Kijima T, Aoe K, et al: Clinical Efficacy and Safety of Nivolumab: Results of a Multicenter, Open-label, Single-arm, Japanese Phase II study in Malignant Pleural Mesothelioma (MERIT). Clin Cancer Res, 2019; 25: 5485-5492. 31164373 10.1158/1078-0432.CCR-19-0103

[4] Hino O, Kobayashi E, Nishizawa M, et al: Renal carcinogenesis in the Eker rat. J Cancer Res Clin Oncol, 1995; 121: 602-605. 7559744 10.1007/BF01197777 PMC12200376

[5] Yamashita Y, Yokoyama M, Kobayashi E, Takai S, Hino O: Mapping and determination of the cDNA sequence of the Erc gene preferentially expressed in renal cell carcinoma in the Tsc2 gene mutant (Eker) rat model. Biochem Biophys Res Commun, 2000; 275: 134-140. 10944454 10.1006/bbrc.2000.3280

[6] Chang K, Pastan I: Molecular cloning of mesothelin, a differentiation antigen present on mesothelium, mesotheliomas, and ovarian cancers. Proc Natl Acad Sci USA, 1996; 93: 136-140. 8552591 10.1073/pnas.93.1.136 PMC40193

[7] Kojima T, Oh-Eda M, Hattori K, et al: Molecular cloning and expression of megakaryocyte potentiating factor cDNA. J Biol Chem, 1995; 270: 21984-21990. 7665620 10.1074/jbc.270.37.21984

[8] Liu X, Chan A, Tai C-H, Andresson T, Pastan I: Multiple proteases are involved in mesothelin shedding by cancer cells. Commun Biol, 2020; 3: 728. 33262421 10.1038/s42003-020-01464-5 PMC7708464

[9] Hino O: Hereditary & environmental cancer - Asbestos･mesothelioma clinic and cancer philosophy clinic. Juntendo Med J, 2019; 65: 328-337.

[10] Hino O, Abe M, Han B, Yan Y: In commemoration of the 2018 Mataro Nagayo Prize: A road to early diagnosis and monitoring of asbestos-related mesothelioma. Cancer Sci, 2019; 110: 1518-1524. 30888083 10.1111/cas.14001 PMC6500980

[11] Shiomi K, Miyamoto H, Segawa T, et al: Novel ELISA system for detection of N-ERC/mesothelin in the sera of mesothelioma patients. Cancer Sci, 2006; 97: 928-932. 16776777 10.1111/j.1349-7006.2006.00246.x PMC11158852

[12] Shiomi K, Hagiwara Y, Sonoue K, et al: Sensitive and specific new enzyme-linked immunosorbent assay for N-ERC/mesothelin increases its potential as a useful serum tumor marker for mesothelioma. Clin Cancer Res, 2008; 14: 1431-1437. 18316566 10.1158/1078-0432.CCR-07-1613

[13] Imashimizu K, Shiomi K, Maeda M, et al: Feasibility of large-scale screening using N-ERC/mesothelin levels in the blood for the early diagnosis of malignant mesothelioma. Experimental and Therapeutic Medicine, 2011; 2: 409-411. 22977518 10.3892/etm.2011.225 PMC3440716

[14] Onda M, Nagata S, Mitchell H, et al: Megakaryocyte potentiation factor cleaved from mesothelin precursor is a useful tumor marker in the serum of patients with mesothelioma. Clin Cancer Res, 2006; 12: 4225-4231. 16857795 10.1158/1078-0432.CCR-06-0472

[15] Hassan R, Remaley T, Sampson ML, et al: Detection and quantitation of serum mesothelin, a tumor marker for patients with mesothelioma and ovarian cancer. Clin Cancer Res, 2006; 12: 447-453. 16428485 10.1158/1078-0432.CCR-05-1477

[16] Creaney J, Francis RJ, Dick IM, et al: Serum Soluble Mesothelin Concentrations in Malignant Pleural Mesothelioma: Relationship to Tumor Volume, Clinical Stage and Changes in Tumor Burden. Clin Cancer Res, 2011; 17: 1181-1189. 21177406 10.1158/1078-0432.CCR-10-1929

[17] Tajima K, Hirama M, Shiomi K, et al: ERC/Mesothelin as a marker for chemotherapeutic response in patients with mesothelioma. Anticancer Research, 2008; 28: 3933-3936. 19192652

[18] Mori T, Tajima K, Hirama M, et al: The N-ERC index is a novel monitoring and prognostic marker for advanced malignant pleural mesothelioma. J Thorac Dis, 2013; 5: 145-148. 23585940 10.3978/j.issn.2072-1439.2013.03.03 PMC3621927

[19] Hooper CE, Lyburn ID, J. Searle J, et al: The southwest area mesothelioma and pemetrexed trial: a multicentre prospective observational study evaluating novel markers of chemotherapy response and prognostication. Br J Cancer, 2015; 112: 1175-1182. 25756396 10.1038/bjc.2015.62 PMC4385956

[20] Hirohashi T, Igarashi K, Abe M, Maeda M, Hino O: Retrospective analysis of large-scale research screening of construction workers for the early diagnosis of malignant mesothelioma. Mol Clin Oncol, 2014; 2: 26-30. 24649303 10.3892/mco.2013.197 PMC3915274

[21] Shiomi K, Shiomi S, Ishinaga Y, et al: Impact of renal failure on the Tumor Markers of mesothelioma, N-ERC/Mesothelin and Osteopontin. Anticancer Res, 2011; 31: 1427-1430. 21508397

[22] Therasse P, Arbuck SG, Eisenhauer EA, et al: New guidelines to evaluate the response to treatment in solid tumors. J Natl Cancer Inst, 2000; 92: 205-216. 10655437 10.1093/jnci/92.3.205

[23] Segawa T, Hagiwara Y, Ishikawa K, et al: MESOMARK kit detects C-ERC/mesothelin, but not SMRP with C-terminus. Biochem Biophys Res Commun 2008; 369: 915-918. 18328258 10.1016/j.bbrc.2008.02.114

[24] Rasband WS: ImageJ, U. S. National Institutes of Health, Bethesda, Maryland, USA. http://imagej.nih.gov/ij (Accessed Feb. 10, 2021).

[25] Schneider CA, Rasband WS, Eliceiri KW: NIH Image to ImageJ: 25 years of image analysis. Nat Methods, 2012; 9: 671-675. 22930834 10.1038/nmeth.2089 PMC5554542

[26] Sato T, Suzuki Y, Mori T, et al: Newly established ELISA for N-ERC/mesothelin improves diagnostic accuracy in patients with suspected pleural mesothelioma. Cancer Med, 2014; 3: 1377-1384. 25045139 10.1002/cam4.297 PMC4302688

[27] Yamaguchi N, Hattori K, Oh-eda M, Kojima T, Imai N, Ochi N: A novel cytokine exhibiting megakaryocyte potentiating activity from a human pancreatic tumor cell line HPC-Y5. J Biol Chem, 1994; 269: 805-808. 8288629

[28] Fenton A, Montgomery E, Nightingale P, et al: Glomerular filtration rate: new age- and gender-specific reference ranges and thresholds for living kidney donation. BMC Nephrol, 2018; 19: 336. 30466393 10.1186/s12882-018-1126-8 PMC6249883

[29] Rubin JB: The spectrum of sex differences in cancer. Trends Cancer, 2022; 8: 303-315. 35190302 10.1016/j.trecan.2022.01.013 PMC8930612

